# Plasma Electrolytic Oxidation as a Feasible Surface Treatment for Biomedical Applications: an *in vivo* study

**DOI:** 10.1038/s41598-020-65289-2

**Published:** 2020-06-19

**Authors:** Tárik Okon Braga Polo, William Phillip da Silva, Gustavo Antonio Correa Momesso, Tiburtino José Lima-Neto, Stéfany Barbosa, Jairo Matozinho Cordeiro, Jaqueline Suemi Hassumi, Nilson Cristino da Cruz, Roberta Okamoto, Valentim A. R. Barão, Leonardo P. Faverani

**Affiliations:** 1 Department of Diagnosis and Surgery, Sao Paulo State University - Unesp. School of Dentistry, Rua José Bonifácio, 1193, Araçatuba, ZIP code:, CEP16015-050 Sao Paulo, Brazil; 2 Undergradutate student, Sao Paulo State University - Unesp. School of Dentistry, Rua José Bonifácio, 1193, Araçatuba, ZIP code:, CEP16015-050 Sao Paulo, Brazil; 3 University of Campinas (UNICAMP), Piracicaba Dental School, Department of Prosthodontics and Periodontology, Av Limeira, 901, Piracicaba, São Paulo, CEP13414-903 Brazil; 4 Institute of Biomaterials, Tribocorrosion and Nanomedicine (IBTN), Sao Paulo, Brazil; 5 Technological Plasma Laboratory (LaPTec), Experimental Campus of Sorocaba, Sao Paulo State University-Unesp, Sorocaba, Brazil; 6 Department of Basic Sciences, Sao Paulo State University - Unesp. School of Dentistry, Rua José Bonifácio, 1193, Araçatuba, ZIP code:, CEP16015-050 Sao Paulo, Brazil

**Keywords:** Biological techniques, Health care, Medical research

## Abstract

Objectives: In this *in vivo* animal study, we evaluated the effect of plasma electrolytic oxidation (PEO) coating on the topographic and biological parameters of implants installed in rats with induced osteoporosis and low-quality bones. Materials and methods: In total 44 Wistar rats (*Rattus novergicus*), 6 months old, were submitted to ovariectomy (OXV group) and dummy surgery (SHAM group). After 90 days, the ELISA test was performed and the ovariectomy effectiveness was confirmed. In each tibial metaphysis, an implant with PEO coating containing Ca^2+^ and P^5+^ molecules were installed, and the other tibia received an implant with SLA acid etching and blasting (AC) (control surface). After 42 days, 16 rats from each group were euthanized, their tibias were removed for histological and immunohistochemical analysis (OPG, RANKL, OC and TRAP), as well as reverse torque biomechanics. Data were submitted to One-way ANOVA or Kruskal-Wallis tests, followed by a Tukey post-test; P < 0.05. Histological analyses showed higher bone neoformation values among the members of the PEO group, SHAM and OVX groups. Immunohistochemical analysis demonstrated equilibrium in all groups when comparing surfaces for TRAP, OC and RANKL (P > 0.05), whereas OPG showed higher PEO labeling in the OVX group (P < 0.05). Biomechanical analysis showed higher reverse torque values (N.cm) for PEO, irrespective of whether they were OVX or SHAM groups (P < 0.05). Conclusion: The results indicated that the PEO texturing method favored bone formation and showed higher bone maturation levels during later periods in osteoporotic rats.

## Introduction

Biomedical researchers have worked continually to improve the structural and biological properties of implant surface textures. The aim of improving these properties is to increase the mechanical strength and corrosion resistance of implants and improve their biological response. This response promotes osseointegration by enlarging the surface contact area, effectively and quickly attracting cells of the osteoblastic lineage, especially in regions of lower bone tissue density^1,2^.

Implant surfaces can be treated by several methods, the types most frequently described in the literature being the techniques that only promote roughness on the surface, such as electro-polishing, mechanical polishing and acid treatments. These are sometimes associated with blasting with titanium oxide (TiO_2_) or aluminum oxide (Al_2_O_3_), oxidation and laser irradiation^3–6^. There are also techniques that not only modify the surface roughness, but also allow the addition of substances or ions^3,7^. The literature contains well documented scientific evidence for superior bone repair results in implants with rough surfaces when compared with machined surfaces^1,6,8,9^.

Texturing techniques allow the addition of calcium, phosphorus or hydroxyapatite – elements that are precipitated on the bone matrix – to be incorporated into it, by means of anodization and biomimetic treatment methods^10,11^. Furthermore, the incorporation of other ions such as zinc, manganese, magnesium and silicon by means of plasma electrolytic oxidation (PEO) coating has been shown^6,9,12^. PEO, also known as anodic spark deposition, is a method for implant surface texturing with well stablished *in vitro* data^13,14^. PEO is a simple and promising technique capable of producing bioactive coatings with micropores on implant surfaces^15–17^. Ca^2+^/P^5+^ associated with PEO has been shown to produce increased mesenchymal stem cell proliferation in culture tests. Therefore, the authors encourage further studies to evaluate this process, thus enabling it to be used in the field of implant dentistry^16–18^.

As previously mentioned, the characteristics of PEO have been shown to be promising for dental medical conditions, such as those found in osteoporosis, defined by the World Health Organization as being a condition in which bone mineral density is equal to or less than 2.5 standard deviations below the peak bone mass found in young adults. Whereas, osteopenia or low bone mass, is a condition in which bone mineral density is between 1 and 2.5 standard deviations below the peak bone mass found in young adults^19,20^.

When factors alter the balance of catabolism and anabolism, bone quality decreases, resulting in the development of a pathological condition that compromises the success rate of osseointegrated implants^21,22^. These systemic changes that cause a significant decrease in bone density are not a universal contraindication for rehabilitative treatment, but they reduce implants survival rates^21–23^. In addition to these systemic situations, some anatomical regions of the maxilla and mandible may have poor bone quality that decreases primary stability and contributes to delayed peri-implant bone repair. These conditions have been a challenge to biomedical engineering to develop changes in the dental implant microstructure to allow for better adhesion of osteoblastic phenotype cells while maintaining other important structural characteristics for implant longevity^1,2^.

Therefore, the aim of this study was to evaluate the feasibility of PEO texturing method that incorporates Ca^2+^ and P^5+^ on the surface of Ti-6Al-4V implants in low-density bone, by i*n vivo* evaluation, based on implant surface topography and biological parameters.

## Materials and methods

### Animal experiment

#### Surface treatment through acid etching and blasting (Group AC)

In this study, Ti-6Al-4V implants of the commercial company (Emfils Dental Implants and Prosthetic Solutions, Itu, Sao Paulo, Brazil) were used, manufactured with the dimensions necessary for use in rats (2 mm in diameter and 6 mm long). The implant surfaces were produced by acid etching and blasting as available from the company (nitric acids, neutral detergent, 95% alcohol, drying, aluminum oxide blasting, 99% alcohol, acid nitric acid, neutral detergent, distilled water, 95% alcohol, drying and packaging), representing the Group AC.

#### PEO treated surfaces

To obtain the PEO surface, machined implants were treated by means of a pulsed direct current (DC) power supply (Plasma Technology Ltd., Kowloon, Hong Kong, China). The processing system consisted of a stainless-steel tank with a cooling system (cathode) and the implants (anode), which were completely submerged in the electrolytic solution that was contained in stainless-steel holders. The electrolyte composition and deposition parameters were based on our previous studies^16,17,24^. The electrolytic solution was prepared by dissolving 0.3 M of calcium acetate [Ca(C_2_H_3_O_2_)_2_] (Dinâmica Ltd., Diadema, SP, Brazil) and 0.02 M of glycerophosphate disodium (C_3_H_7_Na_2_O_6_P) (Sigma-Aldrich, St. Louis, MO, USA) in 500 mL of deionized water. A portable conductivity meter (Russell RL060C; Thermo Scientific, MA, USA) was used to measure the electrical conductivity (13.8 mS.cm^−1^) of the PEO solution before use. Firstly, the probe was rinsed with distilled water and air dried before being inserted into the electrolyte, while stirring it gently to obtain a homogeneous measurement. After preparation, the solution had a pH of 5.27. The pulse voltage, frequency, and duty cycle were established at 290 V, 250 Hz, and 60%, respectively. Deposition was carried out for 10 min, with two implants at a time. Then, the treated implants were rinsed with deionized water and air-dried.

#### Characterization of samples

Scanning electron microscopy (SEM, JEOL JSM-6010LA, Peabody, MA) was performed to analyze the surface morphology of AC- and PEO-textured implants, at baseline, and after removal of implants for reverse-torque biomechanical analysis. Energy dispersive spectroscopy (EDS, JEOL JSM-6010LA, Peabody, MA, USA) was used to evaluate the chemical composition of Ti-6Al-4V implants after treatments. Small-volume elemental chemical analyses (around 1 μm^3^) were performed using the EDS technique. The simultaneous observation of the entire X-ray spectrum facilitated rapid qualitative analysis (mapping) of the main constituent elements of the implant surfaces, thus allowing for a comparison of the chemical compositions of the analyzed surfaces. Image J software (National Institute of Health, USA) was used to determine the average pore size and/or compound diameter deposited for each treatment type. The phase composition of the PEO coating was investigated using the X-ray diffractometry (XRD - X’Pert^3^ PRO MRD; PANalytical, The Netherlands) with a θ–2θ configuration in the 20° to 90° range with a step size of 0.01° and CuKα (λ = 1.54056 Å) radiation.

#### Animal experiment

The study was approved by the Ethics Committee on the Use of Animals of the São Paulo State University (UNESP), School of Dentistry, Araçatuba, Brazil (approval number 01040-2016). The procedures involved were performed in accordance with the guidelines imposed by the Committee on the Use of Animal by the São Paulo State University (UNESP), School of Dentistry, Araçatuba, Brazil, international standards. For the study, 44 Wistar rats (*Rattus novergicus*) were used. Among these rats, 32 were 6 months old (Group OXV) (^16^ and Group SHAM^16^), and 12 rats were used for the ELISA test (OXV^6^) and SHAM^6^). The rats, whose weights ranged from 250 to 300 grams, were of a strain kept in the Bioterium (animal facility) of the Department of Surgery and Integrated Clinic.

Throughout the experiment, the animals were kept in cages in an environment with a stable temperature (22 ± 2 °C), controlled light cycle (12 hours of light and 12 hours of darkness), fed with solid food (Producer Activated Feed, Anderson & Clayton Sa- Laboratorio Abbot do Brasil Ltd, Sao Paulo, SP, Brazil) and water ad libitum, except within the period of 12 hours prior to surgical procedures.

#### Osteoporosis induction

Forty-four female rats (*Rattus novergicus albinus Wistar*), 6 months old were randomly divided into two predetermined groups, SHAM and OVX (n = 16 per group). Group SHAM underwent simulated surgery with exposure of the ovaries that were subsequently repositioned in the abdominal cavity.

Rats in Group OVX were submitted to bilateral ovariectomy surgery. These were anesthetized with xylazine hydrochloride (Xylazine - Coopers, Ltd., Brazil) at a dosage of 5 mg/kg and ketamine hydrochloride (Injectable Ketamine Hydrochloride, Fort Dodge, Saúde Animal Ltd., Brazil) at a dosage of 50 mg/kg. They were then immobilized on a surgical board in a lateral position, and a 1-cm incision was made in the flanks, then through the planes of subcutaneous tissue and the peritoneum to gain access to the abdominal cavity. After this, the ovaries and uterine horns were located and then ligated with polyglactin 910 4.0 wire (Vycryl – Johnson & Johnson, New Brunswick, NJ, USA). At this stage, the ovaries were removed. In the next step polyglactin 910 4.0 (Vycryl – Johnson & Johnson, New Brunswick, NJ, USA) was used for suturing the deeper planes and nylon 4-0 (Ethicon – Johnson & Johnson, New Brunswick, NJ, USA) for the superficial plane. Healthy female rats (normal bone density: SHAM) underwent the same procedure, but their uterine and ovarian horns were only surgically exposed without ligation and removal. In accordance with the requirements of the ethics committee, immediate postoperative medication was administered in the form of single-dose intramuscular dipyrone sodium 500 mg/kg and single-dose intramuscular prophylactic antibiotic (veterinary-pentabiotic) therapy at a dose rate of 0.1 mg/kg of animal weight.

Ninety days after the surgical procedures - the period required for onset of osteoporosis, according to the FDA, 6 animals from each group (SHAM and OVX) were euthanized for the purpose of measuring estrogen levels using the ELISA test, which was performed to confirm the effectiveness of the ovariectomy.

#### Estrogen dosage immunoassay

To determine the effectiveness of the ovariectomy, 6 animals from each group (SHAM and OVX) totaling 12 animals were sedated. A median laparotomy was performed on 6 animals of each group and blood samples were collected in heparinized tubes through the inferior vena cava. These samples were centrifuged at 1000 *g* for 15 min at 4 °C and the plasma obtained was stored at 80 °C until analysis. Estradiol levels were measured using 50 μL of plasma sample for each animal and a monoclonal antibody against rat estradiol, according to the manufacturer’s instructions (ELISA kit for mice Wuhan Fine Biotech CO., Wuhan, China, Cat. No. ER1507). The absorbance reading was performed at a wavelength of 450 nm in a microplate reader and the value of the estrogen concentration was expressed in pg/mL.

### Mechanical testing of bone

The mechanical characteristics of the femurs were assessed by the three-point bending test (MZ-500S; Maruto Instrument). The femurs were positioned facing upwards on a platform, resting on two supports, one anterior and one posterior, with the purpose of stabilizing them and preventing them from moving in any way. A vertical force was applied to the central region of the femurs, at a loading rate of 5 mm/min. Deformation and load curves were used to plot graphs with analysis of the maximum load, stiffness and breaking energy (energy dissipated by bone before breaking apart).

### Implant installation

Ninety days after the OVX and SHAM surgeries, the animals underwent anesthesia and trichotomy was performed on both tibias, followed by antisepsis of the incised region with 10% PVPI (Riodeine Degermante, Rioquímica, São José do Rio Preto). An incision was made in the left and right tibial metaphyseal regions and the soft tissue with periosteum was completely detached, exposing the bone to receive the implants.

Ti-6Al-4V AC-surface implants were used as made available from Emfils Dental Implants, and so were the PEO-treated implants containing Ca/P. For this study, the milling was performed with a 1.4-mm diameter spiral cutter mounted on an electric motor (BLM 600, Driller, São Paulo, SP, Brazil) operating at a speed of 1000 rpm under irrigation with an isotonic sodium chloride solution 0.9% (Physiological, Biosynthetic Laborato Biosynthetic Laboratories Ltd, Ribeirão Preto, SP, Brazil) and a contra-angle with 20:1 reduction (3624 N 1:4 angled part, 67RIC 1:4 head, KaVo, Kaltenbach & Voigt GmbH & Co., Biberach, Germany) with locking and initial stability.

Each animal received two implants, one in each tibia. The choice of tibias for the implantation of the control or test group implants was determined through randomization. One envelope contained two papers on which the words “right” and “left” were written. Another envelope contained two papers, one with the word “AC” and the other with “PEO.” Thus, at the beginning of surgery, a researcher who was not involved in the surgical procedure drew papers for each animal to determine which side would be operated on (right or left) and which implant would be used (AC or PEO).

The sutures were performed in planes, initially with polyglactin 910 – VYCRYL 4.0 (Ethicon, Jonhson & Jonhson, Sao Jose dos Campos, Brazil), follow by the superficial plane sutured with Nylon 5.0 (Ethicon, Johnson & Johnson, Sao José dos Campos, Brazil). Forty-two days after implant placement, 32 animals from Groups SHAM (16) and OVX (16) were euthanized for immunohistochemical, histological and reverse torque analysis, according to previous studies^25–29^. The animals were divided into two subgroups for laboratory analysis: 16 (8 SHAM and 8 OVX) animals for histological and immunohistochemical analysis and 16 (8 SHAM and 8 OVX) animals for reverse torque analysis.

### Analysis of peri-implant bone regeneration

#### Laboratory processing of decalcified tissues

For the histological analysis, 8 animals from each group (8 SHAM and 8 OVX) totaling 16, were euthanized by anesthetic overdose (sodium thiopental at 150 mg/kg intraperitoneally) at 42 days after implant placement. Immediately after euthanasia, the tibias with implants were removed from the animals, fixed in formalin and decalcified in EDTA (Ethylenediamine tetra acetic acid) (10%) for 8 weeks. At this time, the implants were removed with a 1.2-mm hex key by using counterclockwise movement. Subsequently, the pieces were dehydrated in a sequence of 70–100% grades of alcohol. After these steps, the samples were diaphanized with xylol and embedded in paraffin. Sections 5 μm thick were cut using a microtome. The even slides were stained with hematoxylin and eosin (HE) and the odd ones were intended for obtaining immunohistochemical reactions.

### Histometric analysis

After laboratory processing and staining the slides with HE (MERCK & Co., Inc., New Jersey, United States), they were photomicrographed using an optical microscope (Leicar DMLB, Heerbrugg, Switzerland) coupled to a capture camera (Leicar DC 300 F Microsystems Ltd, Heerbrugg, Switzerland) stored in TIFF format. The analyses were performed using Image J software (Processing Software and Image Analysis, Ontario, Canada), of which the “free hands” tool, was used to measure the area of newly formed bone (NBF) in the region of interest - the most central loop of the implant - in pixels.

### Immunohistochemical analysis

For immunohistochemical reactions, hydrogen peroxide was applied to the slides to inhibit the peroxidase activity. The slides were recovered by antigen recovery using a phosphate citrate buffer (pH 6.0). The goat-produced primary polyclonal antibodies was used against tartrate resistant phosphatase (TRAP (Thrombospondin-Related Adhesive Protein**)**; SC 30832; Santa Cruz Biotechnology, Dallas, TX, USA), osteoprotegerin (OPG (Osteoprotegerin); SC 21038; Santa Cruz Biotechnology, Dallas, TX, USA), kappa B-ligand nuclear factor activating receptor (RANKL (Receptor activator of nuclear factor kappa-Β ligand); SC 7627; Santa Cruz Biotechnology, Dallas, TX, USA) and osteocalcin (OC (osteocalcin); SC 18319; Santa Cruz Biotechnology, Dallas, TX, USA) to analyze bone remodeling (OPG and RANKL), mineralization (OC), and osteoclast activity (TRAP) respectively.

Rabbit anti-goat biotinylated secondary antibody (Pierce Biotechnology) was used. Avidin and biotin (PK 6100; elite kit, Vector Laboratories) were used as amplifiers and diaminobenzidine (DAKO, Denmark, SC, USA) as a chromogen. After this step, Mayer hematoxylin counterstaining was performed. The expression of proteins was semi-quantitatively evaluated (ordinal qualitative analysis) by assigning scores according to the intensity and the number of cells labeled in the bone repair. The images were captured with an optical microscope (LEICAR DMLB, Heerbrugg, Switzerland), and analysis was performed using scores that represent the absence of labeling (0), light labeling (1), moderate labeling (2) and intense labeling (3). Diaminobenzidine markings were considered positive, and care was taken to perform negative controls to evaluate the specificity of the antibodies^26,27,29–31^.

#### Biomechanical implant testing (Reverse Torque)

For biomechanical analysis, the tibias of 8 animals from each group (SHAM and OVX) were stabilized immediately after euthanasia, and a digital torque wrench fitted with a 1.2-mm digital hexagon key was adapted to the implant hexagon. An anticlockwise movement was performed until the implant rotated in the peri-implant bone and was completely displaced. The digital torque data were obtained in N.cm.

#### Statistical analysis

All results were submitted to the normality test (Shapiro-Wilk) with a significance value of P < 0.05 using the statistical program Sigmaplot 12.0 (Exakt Graphs And Data Analysis, San Jose, California, USA). The NBF parameter was subjected to two-way ANOVA statistical analysis, considering the first factor of variation as being the animals (2 levels: SHAM vs OVX) and the second factor, the implant surface (AC vs PEO). For comparisons with statistical significance, the Holm-Sidak post-test was applied. The immunohistochemical analysis of the protein expression values (OPG, RANKL, OC and TRAP) and implant surface data were compared for each experimental group the by Kruskal-Wallis test and a Dunn post-test (P < 0.05). For reverse torque analysis (SHAM and PEO), a T test was applied (P < 0.05).

## Results

### Scanning electron microscopy (SEM) and dispersive energy spectroscopy (EDS)

**Figure 1:**
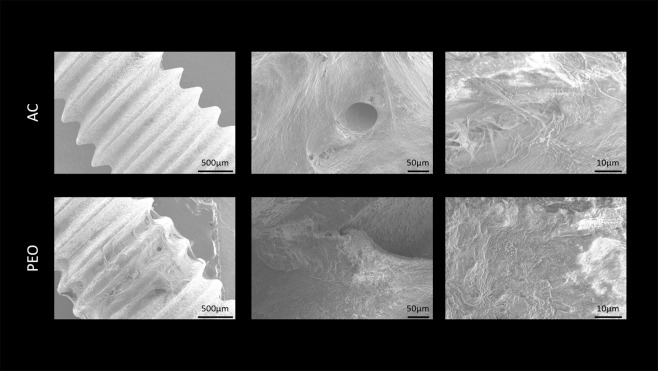
Scanning electron microscopy (SEM) representative image at 50×, 300×, and 2000× magnifications of Ti-6Al-4V alloy implants with AC and PEO textured surfaces after reverse torque (biomechanics: implant removal).

**Figure 2:**
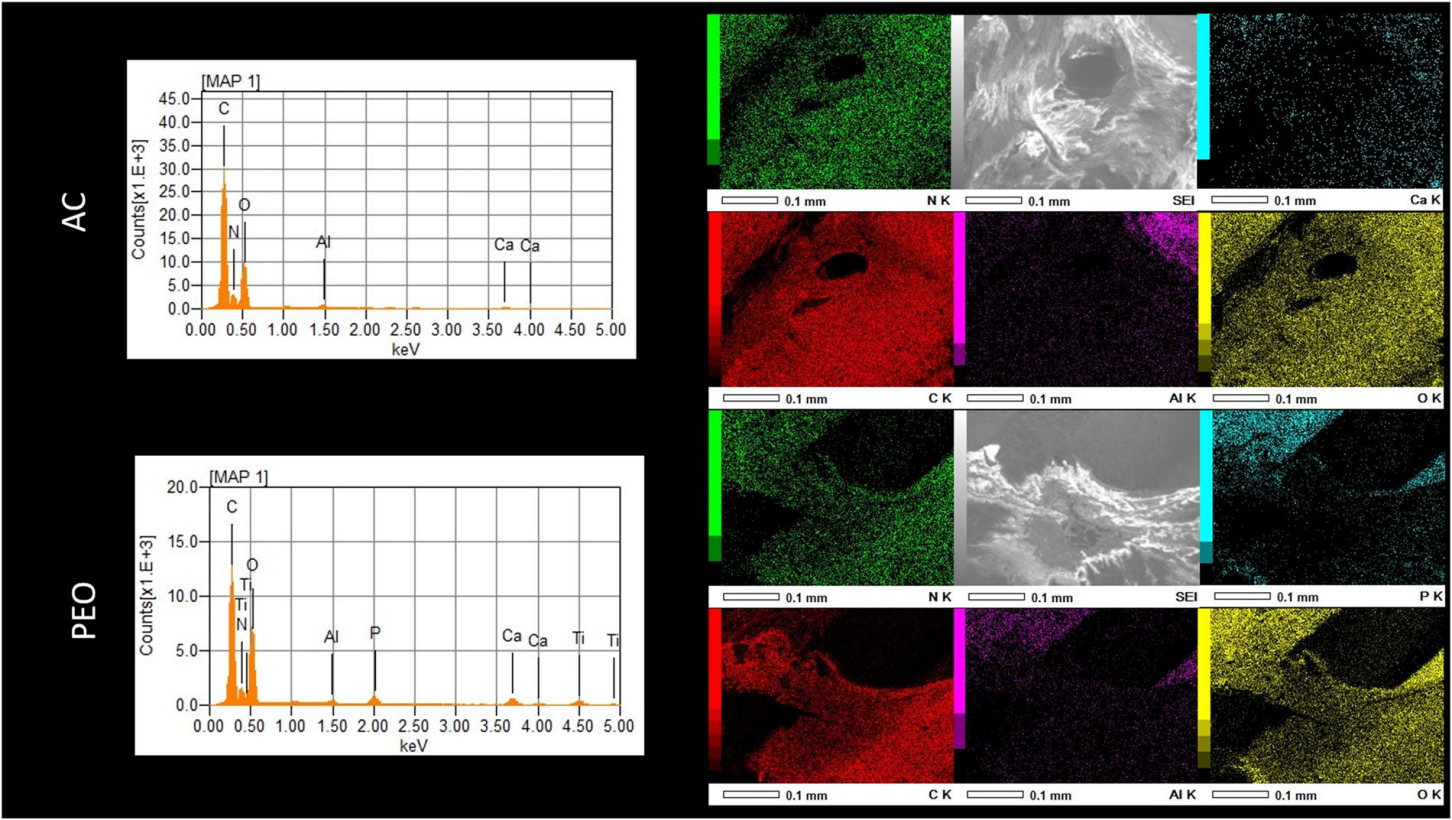
Graphic representation of chemical elements identified by EDS in Groups AC and PEO. Representative images of SEM scanning electron microscopy and color map of Ti-6Al-4V alloy implants with surfaces textured by AC and PEO.

**Figure 3:**
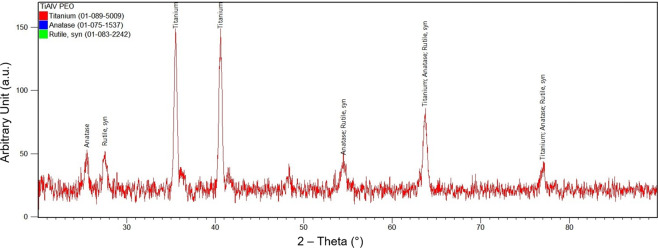
X-ray diffraction patterns obtained from the PEO-treated surface.

Irrespective of the increase in magnitude evaluated by SEM (300× or 2000×), the representation of photomicrographs obtained for the test group (PEO) showed porous surfaces with visually homogeneous pores and geometry similar to that of a volcano. Elemental chemical composition mapping of this surface (PEO) using the EDS technique confirmed the presence of Ti^2+^, Al^2+^, and V into the specimens, as well as the incorporation of Ca^2+^ and P^5+^ (Figs. 1 and 2). Regarding the coating phase composition obtained by XRD (Fig. 3), it was identified the crystalline structures anatase and rutile.

### Estrogen dosage immunoassay

Estrogen dosage after ovariectomy (OVX) and SHAM procedures was evaluated. A statistical difference was observed between the studied groups (OVX 8.3280 with a standard deviation of 2.183 and SHAM 58.19 with a standard deviation of 19.00).

### Mechanical testing of bone

The biomechanical test showed a statistical difference when comparing the force of groups (OVX and SHAM) in newtons relative to fracture (P = 0.018; in the amount of energy required for fracture (P = 0.022), and in stiffness (P = 0.002) (Table 1).

**Table 1:** Average and standard deviation data from the Mechanical Test of Bone for Groups OVX and SHAM femur samples.

Group	Mean Breaking Force (N)	Rigidity (KN/m)	Energy (mJ)
OVX	47.01 ± 11.394	75.57 ± 12.258	16.38 ± 7.151
SHAM	67.35 ± 7.291	117.88 ± 15.372	25.63 ± 3.235

### Histological parameters (HE staining)

**Figure 4:**
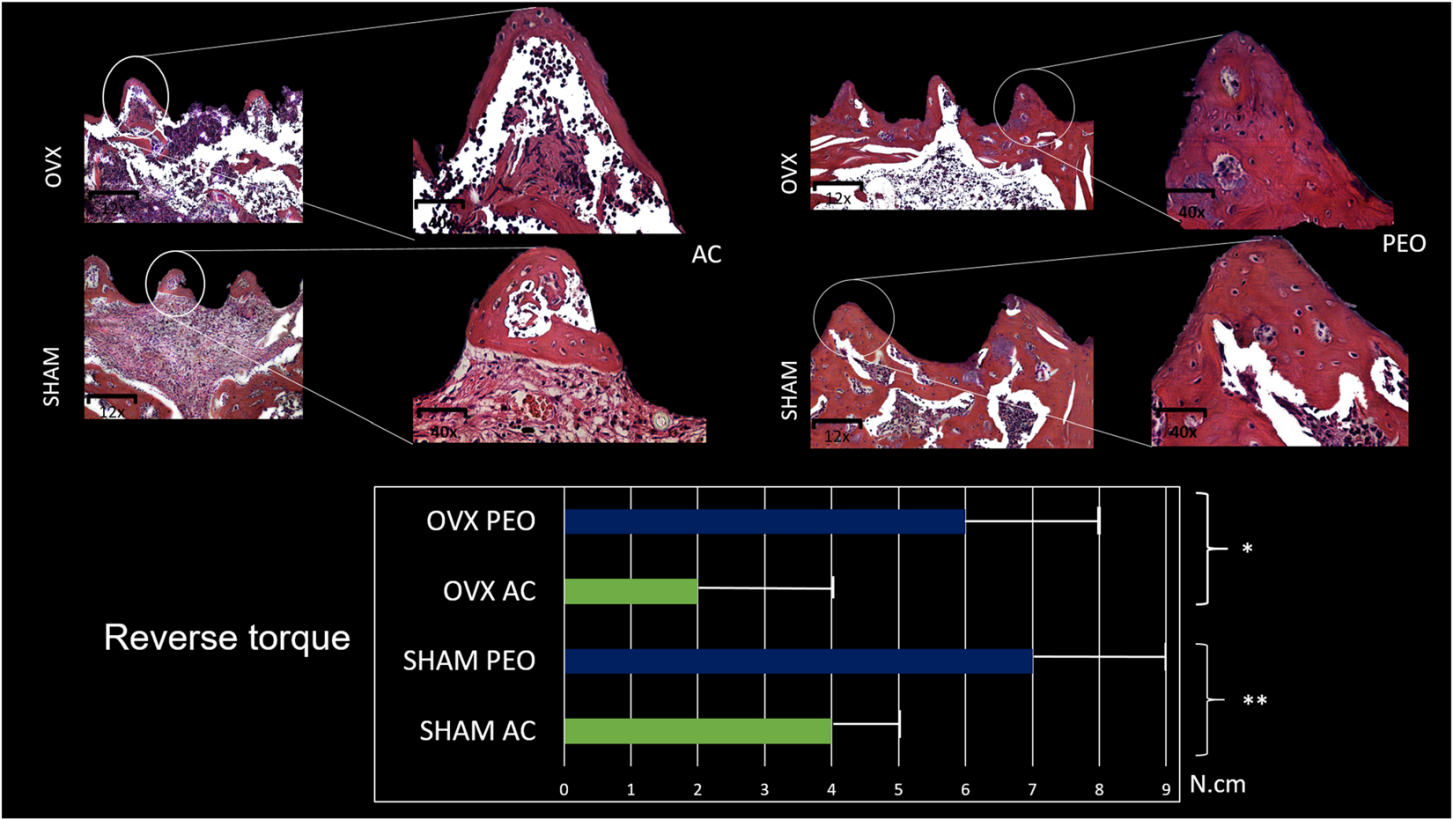
Photomicrograph, representing area of interest of experimental groups (SHAM and OVX) considering implant texturing methods (AC and PEO) in the histological analysis by Hematoxylin and Eosin (HE) staining at 12× and 40× magnifications. Graphical representation of mean values and standard deviation (N.cm) for reverse torque of experimental groups (SHAM and OVX). *OVX group: surface treatment comparison (AC and PEO) P < 0.05. **SHAM group: surface treatment comparison (AC and PEO) P < 0.05. Higher values were observed for PEO surface in both analyses, showing higher bone formation values, consequently higher reverse torque values.

Histological photomicrographs showed that in the interface regions corresponding to the implant threads, newly formed bone tissue was present in the experimental groups analyzed (SHAM and OVX). The AC groups for Groups SHAM and OVX rats showed new bone formation in the higher portions (peaks) of the bone–implant interface and increase in organized connective tissue formation in the lower portions (valleys) of the bone–implant interface. When compared with the AC group, the PEO group showed better bone tissue formation and maturation in the peaks and valleys and little presence of connective tissue for Groups SHAM and OVX (Fig. 4).

### Histometric analysis

**Figure 5:**
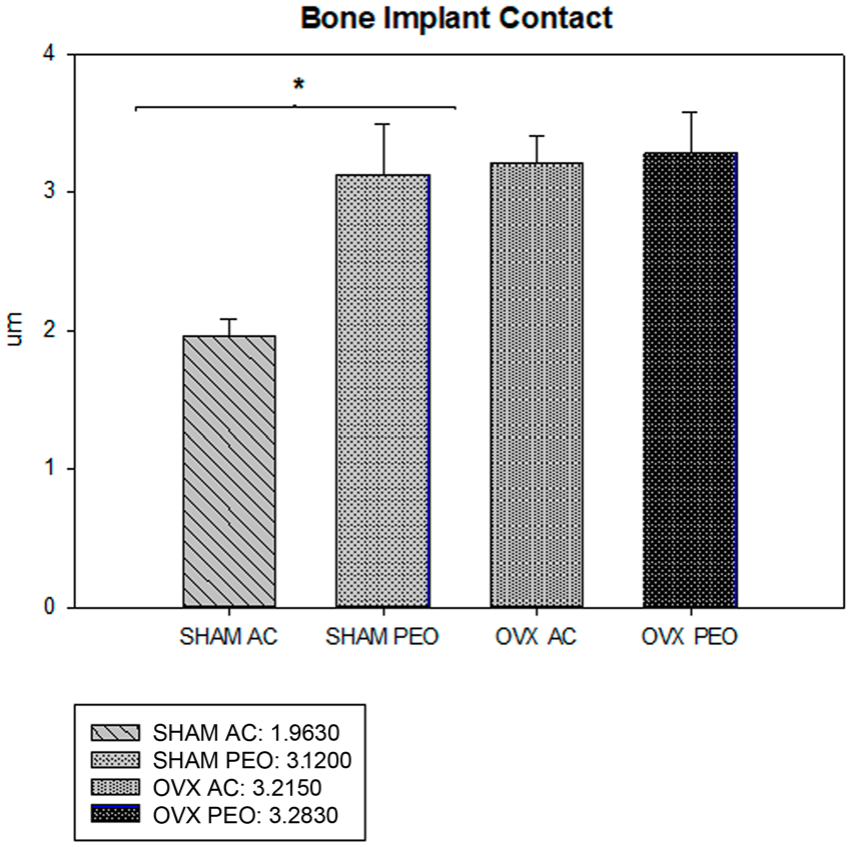
Average and standard deviation data from the histometric analysis (Bone Implant Contact). * denotes P < 0.05.

**Figure 6:**
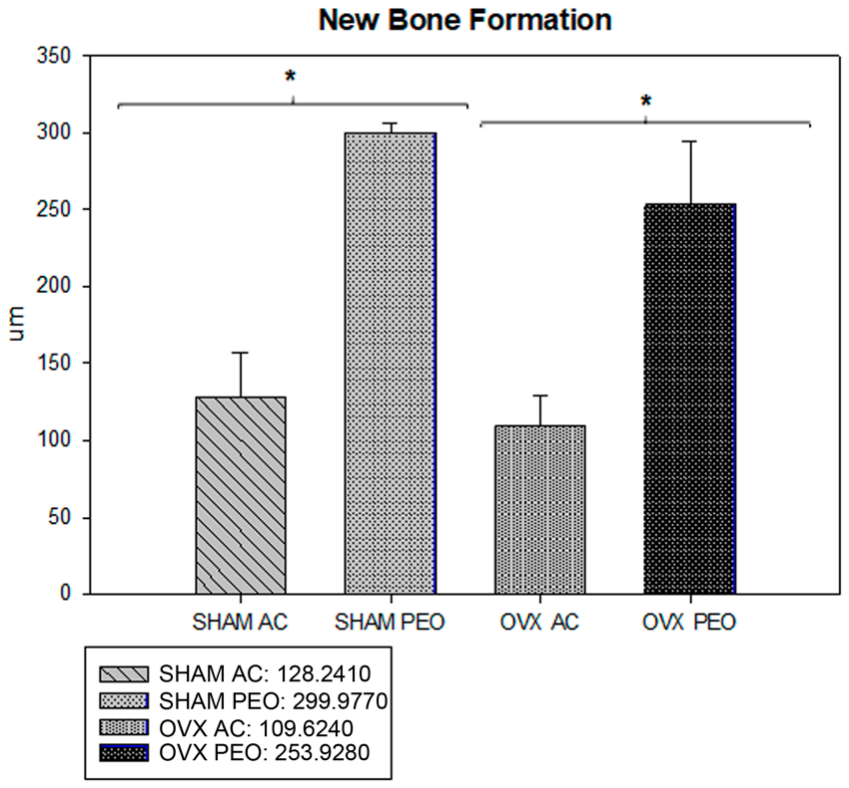
Average and standard deviation data from the histometric analysis (New Bone Formation). * denotes P < 0.05.

New bone formation in the most central loop of the implants showed the following values (mean +SD) (unit: pixel^2^): for Group SHAM AC, 128.2410  ± 28.82; for Group SHAM PEO, 299.9770   ± 6.68; OVX AC 109.6240  ± 19.77, and for Group OVX PEO 253.9280  ± 41.13 (Figs. 5 and 6). These interactions showed a statistical difference in the comparison between the test surfaces (AC vs PEO) (P = 0.001), for both groups of animals (SHAM and OVX). There was no statistical difference in the analysis of the AC surface when comparing the groups (SHAM vs OVX; P = 0.42). There was no statistical difference in the analysis of the PEO surface when comparing Groups (SHAM vs OVX; P = 0.07).

### Immunohistochemical parameters

Group SHAM AC: OPG (1) – There was light marking on bone tissue cells near the implant threads. RANKL (1–2) – There were more markings than those observed for OPG, but these could not be characterized as moderate. OC (1) – There were light, discrete markings on bone tissue cells. TRAP (1–2) – Labeling for this protein was not moderate, but there were a significant number of positively labeled osteoclasts.

Group SHAM PEO: OPG (1) – There was light marking near the bone tissue cells near the implant threads. RANKL (2) – There was moderate marking and the presence of this protein was shown next to the bone tissue under repair. For OC (1-2) and TRAP (2), moderate marking indicated the presence of osteoclasts in bone resorption activity.

Group OVX AC: OPG (0-1) – There was very discreet marking for this protein. RANKL (1) - OC (0-1) – There was very discreet marking for this protein in the analyzed field; TRAP (1-2).

Group OVX PEO - OPG (2) – There was moderate labeling for this protein next to osteoblasts near the implant threads. RANKL (0-1) – There were light to absent marking at the bone–implant interface. OC (1-2) – There was mild to moderate osteocalcin labeling indicating the presence of this protein in bone tissue under repair. TRAP (1-2) – There was low to moderate labeling for resorptive osteoclasts.

**Figure 7:**
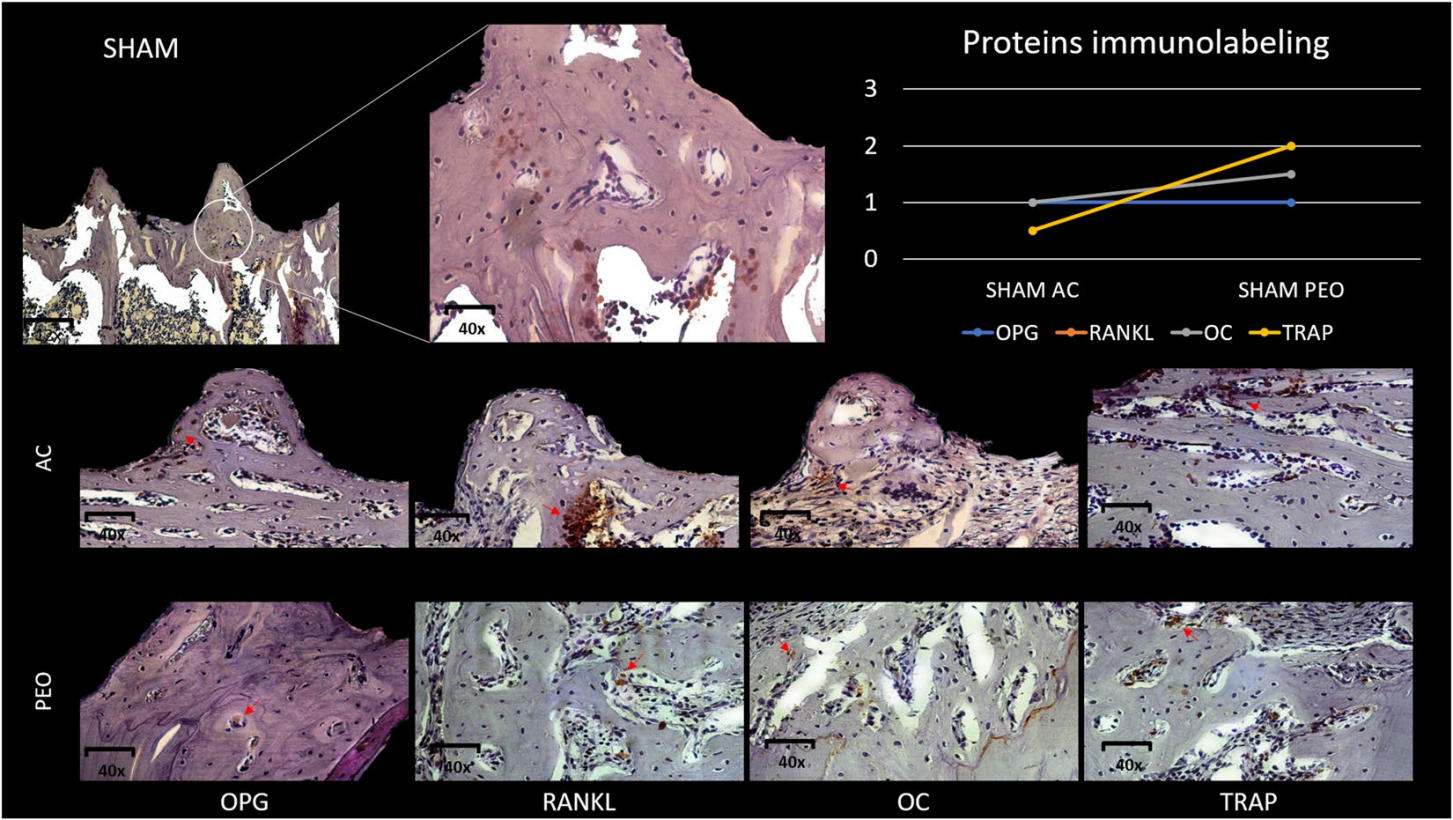
Representative photomicrograph, with increase in magnification of 12× and 40× showing area of interest. Photomicrographs of experimental Group OVX considering texturing methods (AC and PEO), representing positive immunostaining (OPG, RANKL, OC and TRAP proteins), in which similar immunostaining can be observed between two types of surfaces, but only OPG showed the highest immunostaining intensity value (OPG PEO > OPG AC (P < 0.05)). Graphical representation of immunohistochemical analysis scores attributed to OPG, RANKL, OC and TRAP proteins for Group OVX (AC and PEO).

**Figure 8:**
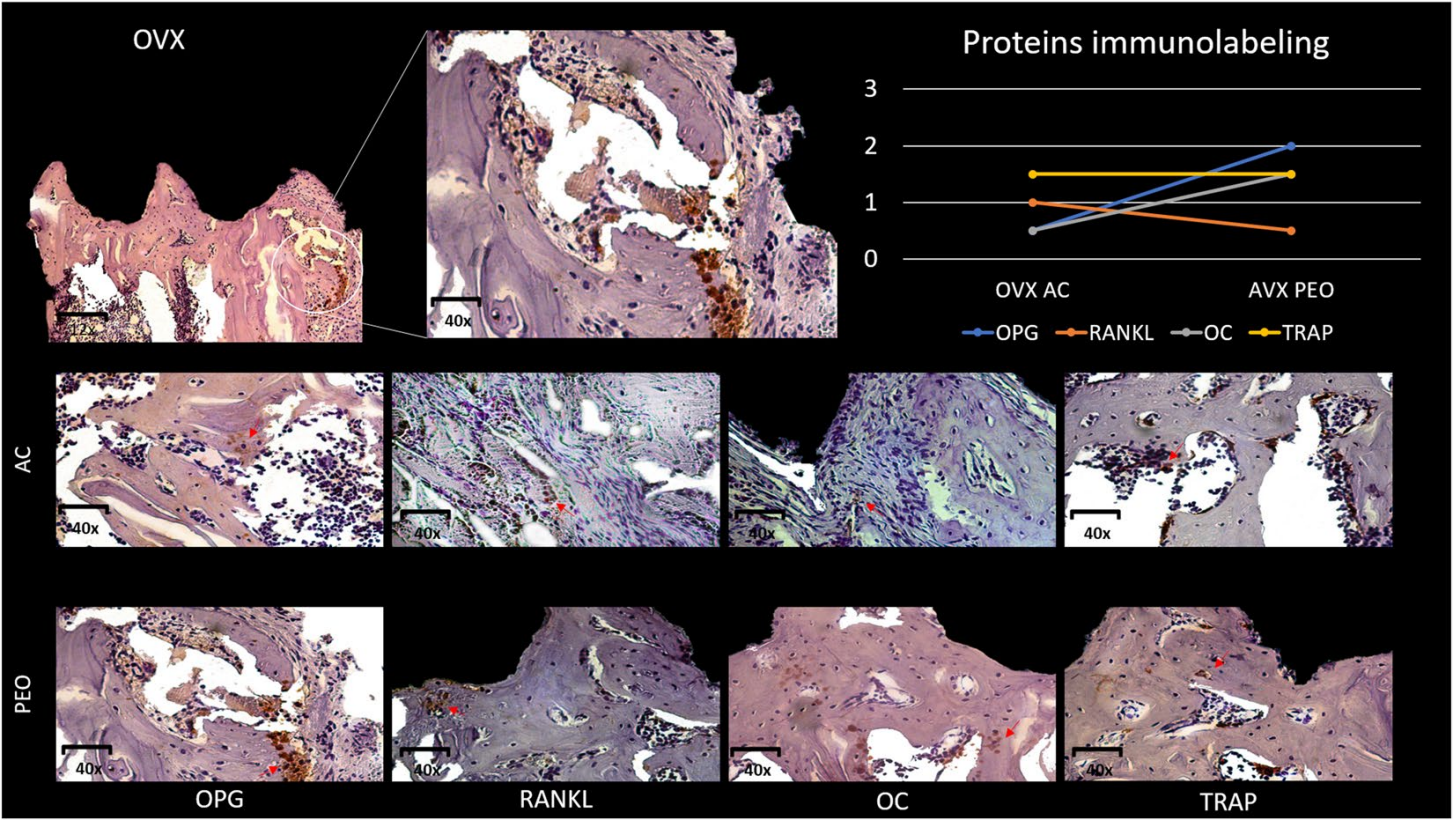
Representative photomicrograph, with increase in magnification of 12× and 40× showing area of interest. Photomicrographs of experimental Group OVX considering texturing methods (AC and PEO), representing positive immunostaining (OPG, RANKL, OC and TRAP proteins), in which similar immunostaining can be observed between two types of surfaces, but only OPG showed the highest immunostaining intensity value (OPG PEO > OPG AC (P < 0.05)). Graphical representation of immunohistochemical analysis scores attributed to OPG, RANKL, OC and TRAP proteins for Group OVX (AC and PEO).

For OPG, only the OVX AC vs. OVX PEO interaction showed statistical significance (P < 0.05) by the presence of a higher level of OVX PEO labeling. For the others, the labeling for this protein were similar (P > 0.05). For RANKL, Group SHAM PEO showed higher immunostaining intensity compared with Group SHAM AC (P < 0.05), however for the other comparisons, the labeling for this protein were similar (P > 0.05). For OC, the statistical comparisons showed no significant difference (P = 0.07), however the immunostaining for the PEO groups (SHAM and OVX) was slightly more moderate in comparison with the groups, in which, most samples were shown to be slightly marked. Some samples from Group OVX AC were even unmarked. For TRAP, all comparisons were similar among the groups (P = 0.39) (Figs. 7 and 8).

### Biomechanical implant testing (Reverse Torque)

In the intragroup analysis (OVX and SHAM), the reverse torque values (mean + SD) (unit: N.cm) were higher for the PEO groups (SHAM = 7.25  ±  1.70; OVX = 5.75   ± 1 1.70) compared with the AC groups (SHAM = 4.00  ± 0.81; OVX = 2.00  ± 1.40) (P < 0.05) (Fig. 4).

## Discussion

The aim of changing the surface topography of osseointegrated implants is to improve their mechanical, corrosion and tribocorrosion properties in an electrolytic oral environment and enhance the biological responses of bone tissue repair. Recent studies have shown that as occurs with other materials submitted to physiological conditions, exposure of implants to mechanical and biological factors can impair their survival and success rates^32^.

Previous studies of PEO surface characterization have shown that an oxide layer is produced on surfaces treated by means of this method, which provides increased wear and corrosion resistance, thermal protection and the possibility of good adhesion of important ions to the surface. In addition, the association of Ca^2+^ and P^5+^ contributed to osseointegration. Herein, the incorporation of Ca^2+^ and P^5+^ showed a crystalline structure containing anatase and rutile, as well as large pores with a volcano-like appearance, as proven by SEM and showed in other studies^2,13,14,16,33–35^.

This study aimed to evaluate the action of PEO with calcium and phosphorous incorporated on the implant surface, by using an osteoporotic rat model to simulate low-quality bone. Thus, the selection of the SHAM and OVX experimental groups revealed that for evaluating the tested biomaterial (AC or PEO), the osteoporotic model promoted by the OVX group showed a significant reduction in estrogen levels when compared with the SHAM groups (P < 0.05), as indicated by the ELISA method, which affected the reparative events. Besides that, the biomechanical test performed by the EMIC machine showed that the SHAM group had a higher rigidity to fracture compared to the OVX group (P < 0.05), with results very similar to the Lucinda *et al*.^36^.

The OVX model was characterized by bone loss and increased osteoclastogenic activity when compared with osteoblastogenesis, in addition to more critical reparative results. This allowed for better evaluation of the biomaterials and topographic changes, as proposed in this study. The results obtained in low-quality bone were promising when considering the dental–medical spectrum, where critical bone conditions are challenging in the context of bone repair.

The histological parameters indicated that there was better bone formation and maturation with the PEO implants compared with AC surface in the SHAM and OVX experimental groups. Anitua *et al*.^37^ evaluated the role of incorporating Ca^2+^ into implants surface on osseointegration using animal models without changes in bone metabolism. They observed a higher stimulation of platelet adhesion and activation and provisional matrix formation, with significantly more osseointegration with respect to unmodified controls. Besides the association of bioactive elements such as Ca^2+^ and P^5+^, the improved bioactivity of PEO surfaces, even for low-density bone, may be due to its crystalline composition that is not commonly observed for acid-etched and sandblasted surfaces. For instance, the presence of anatase have proved to enhance the surface wettability and, therefore, its ability to adsorb components from body fluids (proteins, cells, ions) that may improve implant osseointegration. In fact, TiO_2_ crystalline structures have shown greater ability to stimulate cell proliferation and cortical bone growth^38,39^.

The histological results corroborated the immunohistochemical findings and indicated that there were differences between the groups when the turnover of bone tissue represented by OPG and RANKL was considered. Group OVX PEO showed significantly higher values for OPG protein expression compared with Group OVX AC group. Relative to RANKL protein expression, the lowest concentration was also present in Group OVX PEO, suggesting that the PEO surface stimulated increased bone deposition even in low-density bone tissues. The promising results found in the OPG–RANKL system were apparent when we analyzed the OC protein expression that showed higher values for OVX PEO. Finally, when we analyzed TRAP, we could observe that all groups presented quite similar values for this protein expression, irrespective of the studied surface and the evaluated bone quality.

As regards the biomechanical analysis, the reverse torque results indicated higher removal torque of the implants with the PEO surface in all groups (SHAM > OVX) and lower torque values on the AC surface. Group OVX had the lowest values (SHAM > OVX), which suggests that PEO surfaces provided better biological and clinical responses when compared with AC surfaces. Hao *et al*.^40^ performed biomechanical tests on animals without systemic changes and compared surfaces treated with TiO_2_ blasting and surfaces with anodic oxidation. Their results indicated the superiority of PEO over implants with TiO_2_ blasting. The results were corroborated by He *et al*.^41^, who also performed biomechanical tests on implants installed in rabbit jaws. They observed that the PEO, irrespective of the incorporated elements (Zn^2+^ or Ca^2+^/P^5+^), showed higher bone adhesion values when compared with implants with TiO_2_-treated surfaces. These results indicated that the characteristics of PEO increased the surface area allowing for higher interaction between the implants and bone tissue, favoring cell development and maturation. Thus, it can be stated that surface treatment promoted a higher degree of adhesion between the bone structure and implant, creating a system that facilitated improved distribution of forces that led to good osseointegration and long-term predictability.

The addition of Ca^2+^, P^5+^ and Zn^2+^ to the surface treatment through PEO was capable of optimizing bone formation and remodeling and reducing the osseointegration period by facilitating better bond strength^41^. Studies have shown that the addition of molecules such as Mg^2+^, Al^2+^, Ti^2+^, Ca^2+^ and P^5+^ to commercially pure titanium through the PEO method provided improved corrosion and wear protection, and the best initial cellular response was observed with Ca^2+^ and P^5+^ compared with other elements due to better bioactivity and biocompatibility^42^. According to Zaporozhets *et al*.^43^, the addition of these elements allows better control of tissue inflammatory responses by changing the leukocyte response process, which was more evident in neutrophils in contact with the Ti-6Al-4V surface^43^.

Given the limitations of this *in vivo* study, the results were enlightening and promising, and the study of bioactivity in low-quality bone tissue implants, as performed in this research, is unrelated to osteoporosis treatment. Previous studies^27,44^ have demonstrated that systemic treatment helped the molecular responses of bone tissue biology. However, even in very critical bone metabolism (OVX) situations, the PEO-textured surface was able to exhibit reparative characteristics favorable to osseointegration. Even with such favorable results, further studies are needed to define the long-term behavior of this surface.

Therefore, it could be concluded that the PEO surface method optimized peri-implant repair, promoted a better cellular response and improved bone microarchitecture in osteoporotic rats.

## References

[1] Albrektsson T, Wennerberg A (2004). Oral implant surfaces: Part 1 - Review focusing on topographic and chemical properties of different surfaces and *in vivo* responses to them. Int J Prosthodont.

[2] Faverani, L. P. *et al*. Effect of bleaching agents and soft drink on titanium surface topography. *J Biomed Mater Res B Appl Biomater*. **102**, 22–30 (2014). 10.1002/jbm.b.32949 23661581

[3] Lin A, Wang CJ, Kelly J, Gubbi P, Nishimura I (2009). The role of titanium implant surface modification with hydroxyapatite nanoparticles in progressive early bone-implant fixation *in vivo*. Int J Oral Maxillofac Implants.

[4] Trisi P (2003). Bone-implant contact on machined and dual acid-etched surfaces after 2 months of healing in the human maxilla. J Periodontol.

[5] Oliveira NT (2013). Biomedical Ti-Mo alloys with surface machined and modified by laser beam: biomechanical, histological, and histometric analysis in rabbits. Clin Implant Dent Relat Res.

[6] Queiroz TP (2013). Commercially pure titanium implants with surfaces modified by laser beam with and without chemical deposition of apatite. Biomechanical and topographical analysis in rabbits, Clin Oral Implants Res.

[7] Gupta A, Dhanraj M, Sivagami G (2010). Status of surface treatment in endosseous implant: a literary overview. Indian J Dent Res.

[8] Zechner W (2003). Osseous healing characteristics of three different implant types - A histologic and histomorphometric study in mini-pigs. Clinical oral implants research.

[9] Souza FA (2013). Comparative *in vivo* study of commercially pure Ti implants with surfaces modified by laser with and without silicate deposition: biomechanical and scanning electron microscopy analysis. J Biomed Mater Res B Appl Biomater.

[10] Ferreira Ribeiro C (2016). Initial oral biofilm formation on titanium implants with different surface treatments: An *in vivo* study. Archives of oral biology.

[11] Mendes MWD, Agreda CG, Bressiani AHA, Bressiani JC (2016). A new titanium based alloy Ti-27Nb-13Zr produced by powder metallurgy with biomimetic coating for use as a biomaterial. Mat Sci Eng C-Mater.

[12] Luo W (2015). [Effect of Zinc Doped Calcium Phosphate Coating on Bone Formation and the Underlying Biological Mechanism]. Sheng Wu Yi Xue Gong Cheng Xue Za Zhi.

[13] Krzakala AK-KA, Simka W (2013). Application of plasma electrolytic oxidation to bioactive surface formation on titanium and its alloys. RSC Adv.

[14] Laurindo CA, Torres RD, Mali SA, Gilbert JL, Soares P (2014). Incorporation of Ca and P on anodized titanium surface: Effect of high current density. Mater Sci Eng C Mater Biol Appl.

[15] Akatsu T (2013). Multifunctional porous titanium oxide coating with apatite forming ability and photocatalytic activity on a titanium substrate formed by plasma electrolytic oxidation. Mater Sci Eng C Mater Biol Appl.

[16] Marques Ida S (2015). Incorporation of Ca, P, and Si on bioactive coatings produced by plasma electrolytic oxidation: The role of electrolyte concentration and treatment duration. Biointerphases.

[17] Marques ID (2015). Electrochemical behavior of bioactive coatings on cp-Ti surface for dental application. Corros Sci.

[18] Nagay BE (2019). Visible-Light-Induced Photocatalytic and Antibacterial Activity of TiO2 Codoped with Nitrogen and Bismuth: New Perspectives to Control Implant-Biofilm-Related Diseases. ACS Appl Mater Interfaces.

[19] WHO ScientificGroup. Prevention and Management of Osteoporosis [Internet]. Geneva: World Health Organization; 2003 2014 Jan 30]. Disponível em: http://whqlibdoc.who.int/trs/who_trs_921.pdf.

[20] Chen L (2016). Biomechanical Characteristics of Osteoporotic Fracture Healing in Ovariectomized Rats: A Systematic Review. PLoS One.

[21] Hurst D (2014). Evidence unclear on whether Type I or II diabetes increases the risk of implant failure. Evid Based Dent.

[22] He J, Zhao B, Deng C, Shang D, Zhang C (2015). Assessment of implant cumulative survival rates in sites with different bone density and related prognostic factors: an 8-year retrospective study of 2,684 implants. The International journal of oral & maxillofacial implants.

[23] Chrcanovic BR, Albrektsson T, Wennerberg A (2014). Diabetes and oral implant failure: a systematic review. J Dent Res.

[24] Cordeiro JM (2017). Development of binary and ternary titanium alloys for dental implants. Dent Mater.

[25] Ramalho-Ferreira G, Faverani LP, Prado FB, Garcia IR, Okamoto R (2015). Raloxifene enhances peri-implant bone healing in osteoporotic rats. Int J Oral Maxillofac Surg.

[26] Palin LP, Polo TOB, Batista FRS, Gomes-Ferreira PHS, Garcia IR (2018). Junior, A.C. Rossi, A. Freire, L.P. Faverani, D.H. Sumida, R. Okamoto, Daily melatonin administration improves osseointegration in pinealectomized rats. J Appl Oral Sci.

[27] Faverani LP (2018). Raloxifene but not alendronate can compensate the impaired osseointegration in osteoporotic rats. Clin Oral Investig.

[28] Oliveira D (2017). Short term sodium alendronate administration improves the peri-implant bone quality in osteoporotic animals. J Appl Oral Sci.

[29] Yogui FC (2018). increasing the expression of the osteoblastogenesis and mineralization-related proteins and improving quality of bone tissue in an experimental model of osteoporosis. J Appl Oral Sci.

[30] Pedrosa WF (2009). Immunohistochemical, tomographic and histological study on onlay bone graft remodeling. Part II: calvarial bone. Clinical oral implants research.

[31] Manrique N (2015). Hypertension modifies OPG, RANK, and RANKL expression during the dental socket bone healing process in spontaneously hypertensive rats. Clin Oral Investig.

[32] Ogawa ES (2016). Surface-treated commercially pure titanium for biomedical applications: Electrochemical, structural, mechanical and chemical characterizations, Materials science & engineering. C, Materials for biological applications.

[33] Rizwan M, Alias R, Zaidi UZ, Mahmoodian R, Hamdi M (2018). Surface modification of valve metals using plasma electrolytic oxidation for antibacterial applications: A review. J Biomed Mater Res A.

[34] Teker D (2015). Characteristics of multi-layer coating formed on commercially pure titanium for biomedical applications. Mater Sci Eng C Mater Biol Appl.

[35] Aktug SL, Durdu S, Yalcin E, Cavusoglu K, Usta M (2017). Bioactivity and biocompatibility of hydroxyapatite-based bioceramic coatings on zirconium by plasma electrolytic oxidation. Mater Sci Eng C Mater Biol Appl.

[36] Lucinda LMF (2017). Evaluation of the anti-osteoporotic effect of Ginkgo biloba L. in Wistar rats with glucocorticoid-induced-osteoporosis by bone densitometry using dual-energy x-ray absorptiometry (DEXA) and mechanical testing. An Acad Bras Cienc.

[37] Anitua E, Prado R, Orive G, Tejero R (2015). Effects of calcium-modified titanium implant surfaces on platelet activation, clot formation, and osseointegration. J Biomed Mater Res A.

[38] A. Santos-Coquillata, *et al*., Endzhe Matykinab, *In vitro* and *in vivo* evaluation of PEO-modified titanium for bone implantapplications, pp. 358-368, 2018.

[39] Chung CJ (2013). Plasma electrolytic oxidation of titanium and improvement in osseointegration. J Biomed Mater Res B Appl Biomater.

[40] Hao J (2017). Biological and Mechanical Effects of Micro-Nanostructured Titanium Surface on an Osteoblastic Cell Line *In vitro* and Osteointegration *In vivo*. Appl Biochem Biotechnol.

[41] He J, Feng W, Zhao BH, Zhang W, Lin Z (2018). *In Vivo* Effect of Titanium Implants with Porous Zinc-Containing Coatings Prepared by Plasma Electrolytic Oxidation Method on Osseointegration in Rabbits. The International journal of oral & maxillofacial implants.

[42] Lu X (2016). Plasma electrolytic oxidation coatings with particle additions – A review. Surface and Coatings Technology.

[43] Zaporozhets TS (2017). Biocompatibility of Modified Osteoinductive Calcium-Phosphate Coatings of Metal Implants. Bull Exp Biol Med.

[44] Ramalho-Ferreira, G., Faverani, L. P., Grossi-Oliveira, G. A., Okamoto, T. & Okamoto, R. Alveolar bone dynamics in osteoporotic rats treated with raloxifene or alendronate: confocal microscopy analysis, *J Biomed Opt* **20**(3), 038003 (2015). 10.1117/1.JBO.20.3.038003 25813805

