# Author Correction: Plasma Electrolytic Oxidation as a Feasible Surface Treatment for Biomedical Applications: an *in vivo* study

**DOI:** 10.1038/s41598-020-75951-4

**Published:** 2020-10-28

**Authors:** Tárik Ocon Braga Polo, William Phillip Pereira Silva, Gustavo Antonio Correa Momesso, Tiburtino José Lima-Neto, Stéfany Barbosa, Jairo Matozinho Cordeiro, Jaqueline Suemi Hassumi, Nilson Cristino da Cruz, Roberta Okamoto, Valentim A. R. Barão, Leonardo P. Faverani

**Affiliations:** 1 Department of Diagnosis and Surgery, Sao Paulo State University - Unesp. School of Dentistry, Rua José Bonifácio, 1193, Araçatuba, Sao Paulo ZIP code:, CEP16015-050 Brazil; 2 Sao Paulo State University - Unesp. School of Dentistry, Rua José Bonifácio, 1193, Araçatuba, Sao Paulo ZIP code:, CEP16015-050 Brazil; 3 Piracicaba Dental School, Department of Prosthodontics and Periodontology, University of Campinas (UNICAMP), Av Limeira, 901, Piracicaba, São Paulo CEP13414-903 Brazil; 4 Institute of Biomaterials, Tribocorrosion and Nanomedicine (IBTN), Sao Paulo, Brazil; 5 Technological Plasma Laboratory (LaPTec), Experimental Campus of Sorocaba, Sao Paulo State University-Unesp, Sorocaba, Brazil; 6 Department of Basic Sciences, Sao Paulo State University - Unesp. School of Dentistry, Rua José Bonifácio, 1193, Araçatuba, Sao Paulo ZIP code:, CEP16015-050 Brazil

Correction to: *Scientific Reports* 10.1038/s41598-020-65289-2, published online 19 June 2020

The original version of this Article contained errors in the spelling of the authors Tárik Ocon Braga Polo and William Phillip Pereira Silva which were incorrectly given as Tárik Okon Braga Polo and William Phillip da Silva respectively.

As a result, the Author Contribution statement,

“Tiburtino José de Lima Neto, Stéfany Barbosa and William Phillip Pereira da Silva: These authors were responsible for writing part of the text, such as, introduction, methodology, as well as analyzing and writing the results. Nilson Cristino da Cruz, Jairo Matozinho Cordeiro, Roberta Okamoto: These authors were responsible for Histological analysis and immunohistochemical analysis. Jaqueline Suemi Hassumi: This author performed all immunohistochemistry reactions and collected data. Gustavo Antonio Correia Momesso and Tárik Okon Braga Polo: These authors were responsible for writing the discussion of manuscript. Leonardo Perez Faverani and Valentim Adelino Ricardo Barão: These authors were the idealizers and advisors of this study. Thus, they had a fundamental importance in all steps of preparing the manuscript, performing the review and correcting the results and text.”

now reads:

“Tiburtino José de Lima Neto, Stéfany Barbosa and William Phillip Pereira Silva: These authors were responsible for writing part of the text, such as, introduction, methodology, as well as analyzing and writing the results. Nilson Cristino da Cruz, Jairo Matozinho Cordeiro, Roberta Okamoto: These authors were responsible for Histological analysis and immunohistochemical analysis. Jaqueline Suemi Hassumi: This author performed all immunohistochemistry reactions and collected data. Gustavo Antonio Correia Momesso and Tárik Ocon Braga Polo: These authors were responsible for writing the discussion of manuscript. Leonardo Perez Faverani and Valentim Adelino Ricardo Barão: These authors were the idealizers and advisors of this study. Thus, they had a fundamental importance in all steps of preparing the manuscript, performing the review and correcting the results and text.”

In addition, the author William Phillip Pereira Silva was incorrectly indexed. This error has now been corrected.

Finally, the Acknowledgements section in this Article was incomplete.

“This study was supported by the São Paulo Research Foundation – FAPESP (2016/20297-6).”

now reads:

“The authors would like to thank the São Paulo Research Foundation – FAPESP (2016/20297-6 and 2020/06195-1) for providing the funding.”

These errors have now been corrected in the PDF and HTML versions of the Article.

